# MicroRNA-383-5p regulation of NADPH oxidase 4 alleviates hemorrhagic transformation and improves acute outcomes after endovascular recanalization in acute ischemic stroke

**DOI:** 10.3389/fimmu.2026.1870493

**Published:** 2026-06-24

**Authors:** Qiu-Yi Jiang, Wang Qin, Jin-Kun Zhuang, Ze-Xin Chen, Qian-Yu Chen, Chang-Luo Li, Zhong-Song Shi

**Affiliations:** 1 Department of Neurosurgery, Sun Yat-sen Memorial Hospital, Sun Yat-sen University, Guangzhou, China; 2 RNA Biomedical Institute, Sun Yat-sen Memorial Hospital, Sun Yat-sen University, Guangzhou, China; 3 Nanhai Translational Innovation Center of Precision Immunology, Sun Yat-sen Memorial Hospital, Sun Yat-sen University, Foshan, China; 4 Guangdong Province Key Laboratory of Brain Function and Disease, Sun Yat-sen University, Guangzhou, China

**Keywords:** acute ischemic stroke, hemorrhagic transformation, inflammation, microRNA, NOX4, oxygen-glucose deprivation reoxygenation

## Abstract

**Background:**

Hemorrhagic transformation (HT) following reperfusion therapy is associated with poor outcomes in patients with acute ischemic stroke. Here, we determined the role of microRNA-383-5p (miR-383-5p) in alleviating HT-associated injury using models of middle cerebral artery occlusion (MCAO) and oxygen-glucose deprivation/reoxygenation (OGD/R).

**Methods:**

Two hundred and five hyperglycemic rats were used to establish a model of HT induced by mechanical recanalization after 5 hours of MCAO, followed by 3 and 6 hours of recanalization; then, miR-383-5p agomir was administered intravenously before recanalization. Analysis involved brain water content; hemorrhage severity; infarct volume; blood-brain barrier (BBB) disruption; neuronal apoptosis, reactive oxygen species (ROS) production; along with the expression levels of miR-383-5p, NADPH oxidase 4 (NOX4), interleukin 1 beta (IL-1β), and BBB-associated proteins. The expression of miR-383-5p and NOX4 was also evaluated in neurons after OGD/R, whereas neuronal injury and ROS production were assessed following intervention with miR-383-5p and small interfering RNA.

**Results:**

In the MCAO model, intravenous administration of miR-383-5p agomir increased miR-383-5p and suppressed NOX4 upregulation in peri-infarct tissue and in brain microvascular endothelial cells, neurons, and astrocytes, as demonstrated by immunohistochemical fluorescent staining. Increased levels of miR-383-5p alleviated brain edema, infarct volume, and the severity of hemorrhage; reduced ROS overproduction, neuronal apoptosis, and IL-1β overexpression in astrocytes; and preserved BBB integrity after mechanical recanalization following ischemia, thereby improving acute neurological outcomes assessed at 3 and 6 hours after recanalization. Following OGD/R, reduced miR-383-5p and increased NOX4 expression were detected in neurons. Elevated miR-383-5p levels reduced neuronal injury, ROS overproduction, and NOX4 overexpression in OGD/R-treated neurons. NOX4 was confirmed as a direct target gene of miR-383-5p.

**Conclusions:**

MiR-383-5p alleviated oxidative stress injury, neuronal apoptosis, inflammation, and preserved BBB integrity by regulating NOX4. Therefore, miR-383-5p represents a potential therapeutic target to reduce HT and improve outcomes during the acute phase of recanalization after endovascular treatment for acute ischemic stroke.

## Introduction

Reperfusion therapy with thrombolysis and endovascular thrombectomy can benefit patients with acute ischemic stroke (AIS); however, hemorrhagic transformation (HT) following these treatments is associated with a poor long-term prognosis (1, 2). Recent studies have shown that asymptomatic HT, previously considered a benign phenomenon with the occurrence in one to three patients following endovascular treatment, can also significantly impact functional outcomes (3, 4). In light of this new understanding, developing a neurovascular protective strategy as an effective adjunct to reperfusion treatment is crucial for the alleviation of HT, thereby improving outcomes in AIS.

The mechanisms of oxidative stress, inflammation, and apoptosis are crucial for understanding HT after treatment (2). In the central nervous system, nicotinamide adenine dinucleotide phosphate oxidases (NOXs) are the primary sources of reactive oxygen species (ROS) generation, which play an important role in regulating the redox state of neurovascular cell homeostasis and key processes related to stroke (5). Targeting NOX4, one of the most prevalent of the NOX family in neurons and endothelial cells, is known to reduce cerebral ischemia-reperfusion injury and minimize hemorrhage by alleviating oxidative stress injury and the disruption of the blood-brain barrier (BBB) during AIS (6–8).

MicroRNAs have been recognized as potential biomarkers for diagnosis and prognostic prediction in AIS (9). MicroRNA-383 (miR-383) is one of the most enriched microRNAs in neurons within the brain (10). Furthermore, analysis of clinical blood samples has revealed that the downregulation of miR-383 may be associated with HT in patients with AIS (11). Moreover, reduced levels of miR-383-5p have been detected in peri-infarct tissue obtained from the middle cerebral artery occlusion (MCAO) model with HT induced by mechanical recanalization in our previous study (12). However, the protective role of miR-383-5p in HT has yet to be elucidated.

Recent studies suggest that NOX4 plays an important role in the inflammatory mechanisms of central nervous system diseases (13, 14). NOX4 was among the predicted target genes of miR-383-5p in the miRDB and TargetScan databases. The present study investigated the protective role of miR-383-5p in reducing HT by directly targeting NOX4 and in relieving inflammation, using the MCAO model with HT induced by mechanical recanalization in hyperglycemic rats and the oxygen-glucose deprivation/reoxygenation (OGD/R) model in neurons. The goal of this study was to investigate the potential of miR-383-5p as a novel therapeutic target to alleviate HT and improve acute outcomes following mechanical endovascular reperfusion treatment.

## Materials and methods

### Induction of HT in hyperglycemic MCAO rats by mechanical recanalization

The animal research protocol was approved by the Institutional Animal Care and Use Committee at our institution. The intraluminal filament technique was utilized to occlude the left middle cerebral artery for 5 hours in adult male Sprague-Dawley hyperglycemic rats (8–10 weeks of age, 250–280g in weight) to induce severe cerebral ischemia, as previously reported (7, 15, 16). To create more obvious HT, rats were induced into acute hyperglycemia via intraperitoneal injections of 50% dextrose (6ml/kg) 15 minutes pre-occlusion, maintaining a blood glucose level >16.7 mmol/L. Sustained hyperglycemia during ischemia was achieved via supplemental injections of 50% dextrose (1.5 ml/kg) at 1, 2, 3, and 4 hours post-MCAO. The rats received recanalization at 6 hours after a 5-hour occlusion, with retrieval of the intraluminal filament. All experimental procedures were performed under anesthesia using 5% isoflurane for induction and 2% isoflurane for maintenance.

### Administration of the MiR-383-5p agomir to the HT model

First, 30 hyperglycemic rats were randomly allocated to evaluate miR-383-5p levels in blood and brain tissue across a sham operation group and four 5-hour MCAO groups with various recanalization time points (0, 1, 3, and 6 hours), with six rats per group. Second, 175 hyperglycemic rats were randomly allocated into five groups to elucidate the neurovascular protective role of miR-383-5p intervention: sham operation (n=35), MCAO (n=35), MCAO with microRNA control intervention (n=35), and MCAO receiving miR-383-5p agomir treatment following recanalization at 3 hours (n=35) or 6 hours (n=35). The miR-383-5p agomir or a microRNA control (50 nmol/kg body weight; Gene Pharma) was injected intravenously half an hour before the intraluminal filament was retrieved. After 3 or 6 hours of recanalization, rats were sacrificed to harvest brain tissue and whole peripheral blood samples. For RNA analysis, whole blood was stored in PAXgene Blood RNA tubes (PreAnalytiX GmbH). Peri-infarct tissues were also collected for RNA and protein assays. Brain hemispheres were collected in Optimal Cutting Temperature compound and snap-frozen. Immunohistochemical staining, terminal deoxynucleotidyl transferase dUTP nick end labelling (TUNEL) staining, and ROS measurement were performed using cryosections that were 7 μm thick.

### Assessment of neurological function and infarct volume in the rat MCAO model

Rats were examined and scored for neurological deficits on a scale from 0 to 4 at 3 and 6 hours after reperfusion. The five-point scale was determined as follows: 0, no neurological deficits; 1, failure to extend the contralateral forepaw fully; 2, reduced resistance to lateral push; 3, spontaneous circling to the contralateral side; and 4, absence of spontaneous movement or unconsciousness.

2,3,5-triphenyltetrazolium chloride (TTC) staining was used to evaluate infarct volumes. At 3 or 6 hours after recanalization, rat brains were cut into 6 serial coronal sections, each 2 mm thick, which were then stained with a 2% TTC solution. Infarct volume with edema correction was calculated using the following formula: 100 × (contralateral hemisphere volume-noninfarct ipsilateral hemisphere volume)/contralateral hemisphere volume by ImageJ software.

### Assessment of brain water content in the rat MCAO model

The wet-dry weight method was used to evaluate brain edema. Brain samples were quickly removed and weighed with an electronic analytical balance to determine the wet weight. The tissue was then dried at 100°C for 24 hours and reweighed again to obtain the dry weight. Water content in the brain was then calculated as follows: 100% × (wet weight - dry weight)/wet weight.

### BBB permeability assessment by Evans blue staining

Three hours before sacrifice, the tail veins of experimental rats were injected with Evans blue (EB) solution. The rats were then anesthetized to allow perfusion of the left cardiac ventricle with saline to clear intravascular EB dye. Then, the brain was dissected into ischemic and non-ischemic hemispheres. Trichloroacetic acid was added to each hemisphere to precipitate protein, and the samples were centrifuged. Supernatants were then evaluated for EB absorbance using a spectrophotometer. The absorbance intensity of EB extravasation in each hemisphere was measured to calculate the EB extravasation index, a reflection of BBB permeability.

### Assessment of HT in the rat MCAO model

Hemoglobin content in each hemisphere was assessed using a spectrophotometric assay to quantify cerebral hemorrhage at 3 or 6 hours after mechanical recanalization. The brain was removed and dissected into left and right hemispheres; the hemispheric tissue was then homogenized and centrifuged. Next, the supernatant was mixed with Drabkin’s reagent (Sigma) and incubated. Optical density was then measured by spectrophotometry. Hemoglobin content index was calculated as the hemoglobin content in the ipsilateral hemisphere divided by that in the contralateral hemisphere.

### *In vitro* studies in the neuronal model of OGD/R

Primary mouse cortical neurons were cultured according to an established method (6, 12). In OGD/R experiments, the third-generation neurons were deprived of oxygen and glucose in a hypoxic chamber (95% N_2_ and 5% CO_2_, Stemcell) using glucose-free medium for 2 hours. Then, neurons were reoxygenated in a normoxic chamber with a glucose-containing medium at dynamic time points from 0 to 72 hours. The expression levels of miR-383-5p and NOX4 were determined by RT-PCR.

MicroRNA mimics and inhibitors, small interfering RNA (siRNA), and Lipofectamine 3000 (Invitrogen) were used for transfection. MiR-383-5p mimics and inhibitors were used at concentrations of 50 nM and 100 nM, respectively. Neurons were incubated with miR-383-5p mimics, miR-383-5p inhibitors, or siRNA NOX4 for 48 hours and then underwent 2-hour OGD with reoxygenation at 24 hours. Finally, neurons were collected for RNA assays, ROS measurements, and lactate dehydrogenase (LDH) assays. MiR-383-5p mimics and inhibitors were synthesized by RiboBio. siRNA targeting NOX4 was synthesized by Gene Pharma.

### Quantitative RT-PCR

Quantitative real-time PCR was performed to determine the levels of miR-383-5p and NOX4 in peri-infarct tissue and peripheral blood from the animal model, as well as in neurons following OGD/R. RNA was isolated using the TRIzol reagent, and primers were sourced from RiboBio. For normalization, U6 served as the internal reference for miR-383-5p, whereas β-actin was used for NOX4 mRNA. The expression levels of miR-383-5p and NOX4 were quantified using the 2-ΔΔCT method, and each sample was tested in triplicate.

### Western blotting

Total protein was extracted from peri-infarct tissue obtained from the MCAO model. The levels of NOX4, claudin5, and occludin proteins were then assessed by enhanced chemiluminescence using the following antibodies purchased from Abcam at 1:1000 dilutions: NOX4 (ab133303), claudin5 (ab131259), occludin (ab216327), and β-actin (ab227387). A molecular imager system was used to detect protein expression bands, and each sample was tested in triplicate. The ImageJ software was then used to quantify optical density.

### Immunohistochemical fluorescent staining

Cryosections of brain tissue were stained by immunohistochemistry using the following primary antibodies at a dilution of 1:200: NOX4 (Abcam, ab133303), interleukin 1 beta (IL-1β) (Abcam, ab315084), neuronal nuclear protein (NeuN) as a marker for neuron (Millipore, MAB377), glial fibrillary acidic protein (GFAP) as a marker for astrocyte (Millipore, MAB360), ionized calcium-binding adaptor molecule 1 (Iba1) as a marker for microglia (Abcam, ab283319), and platelet endothelial cell adhesion molecule-1 (CD31) as a marker for brain microvascular endothelial cells (Santa Cruz, sc-376764). The fluorescent secondary antibodies Alexa Fluor 594 (Invitrogen, A11012) or Alexa Fluor 488 (Invitrogen, A21202) secondary antibodies (1:250) were then applied to the sections. 4’,6-diamidino-2-phenylindole (DAPI) was applied to stain the nuclei. A confocal microscope was then used to capture three random images from the peri-infarct region per section. ImageJ software was used to quantify the area of positive fluorescence.

### ROS assays

Next, we measured ROS levels in brain cryosections and in an OGD/R neuronal model using dihydroethidium (DHE) (Sigma, D7008) and 2′,7′-dichlorodihydrofluorescein diacetate (DCFH-DA) (Sigma, D6883) to detect superoxide anion and hydrogen peroxide, respectively. Brain cryosections and neurons from the OGD/R model were incubated with 10 μmol/L of DHE or DCFH-DA for 30 minutes according to the manufacturer’s protocols. Brain sections and neurons were then photographed with a fluorescence microscope to capture three random images per section. The ImageJ software was used to quantify the positive fluorescence area for ROS (superoxide anion and hydrogen peroxide) in the peri-infarct region of the MCAO model and in the neuronal OGD/R model.

### TUNEL assays

TUNEL and NeuN double staining were performed to assess neuronal apoptosis in the peri-infarct region, according to the protocol, using the TUNEL *in situ* cell death detection kit (Roche, 12156792910). Brain sections and neurons were then photographed with a fluorescence microscope to capture three random images per section. The ratio of TUNEL-positive neurons to the total number of neurons was used as an apoptotic index to assess the extent of neuronal apoptosis.

### LDH assays

LDH release in the neuronal OGD/R model was evaluated with an LDH Cytotoxicity Colorimetric Assay Kit in accordance with the manufacturer’s protocol. LDH activity was assessed by measuring absorbance at 490 nm in each group to evaluate neuronal damage.

### Dual-luciferase reporter assay

Finally, dual-luciferase activity technology was used to determine the relationship between miR-383-5p and NOX4. Human embryonic kidney 293T cells were seeded into 24-well plates, and X-tremegene HP (ROCHE) was used for plasmid transfection. MiR-383-5p mimics were transfected into cells with wild-type and mutant NOX4-3’UTR fused to luciferase. Passive Lysis Buffer was added to the wells to lyse cells. A Luciferase Assay Reagent (Promega) was then used to determine firefly luminescence, whereas Stop & Glo Reagent was used to determine Renilla luminescence. The effect of miR-383-5p on the activity of the NOX4 luciferase reporter gene was then determined.

### Statistical analysis

All statistical evaluations were performed using SPSS version 31.0. Data are expressed as mean ± standard deviation. The Shapiro-Wilk test was used to assess normality, and Levene’s test was used to evaluate homogeneity of variance. For comparisons between two groups, normally distributed data were analyzed using the Student’s t-test. For comparisons among more than two groups, normally distributed data were analyzed using one-way analysis of variance (ANOVA) followed by Bonferroni-adjusted *post hoc* multiple comparisons. Sample size (n = 5–6 per group for *in vivo* experiments; n = 3 independent experiments for *in vitro* studies) was determined based on previous publications. For *in vivo* experiments, each group included 5–6 rats (biological replicates), and each animal was processed independently. For *in vitro* experiments (the OGD/R model), each group included three independent cell culture preparations (biological replicates), and technical replicates were averaged to obtain a single value per biological replicate. A p-value < 0.05 was considered statistically significant.

## Results

### Reduced levels of miR-383-5p in the brain and blood after mechanical recanalization were reversed by the intravenous administration of miR-383-5p agomir

**Figure 1:**
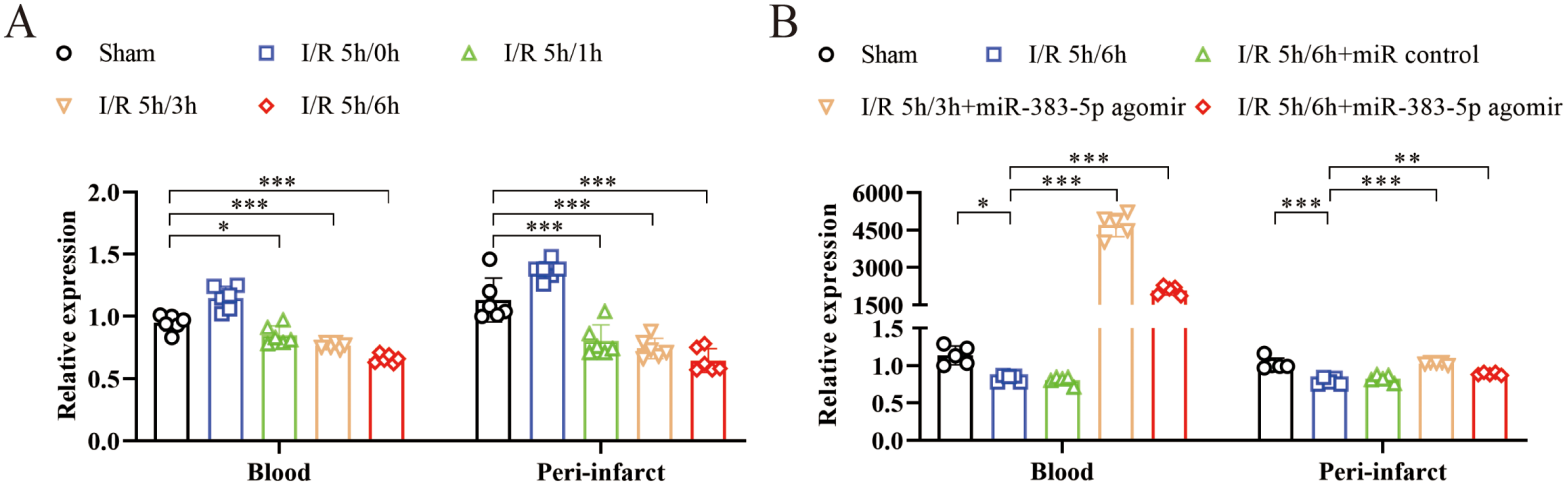
The intravenous administration of miR-383-5p agomir restored the reduced levels of miR-383-5p expression in a hyperglycemia-associated, recanalization-induced hemorrhagic transformation model. **(A)** the expression of miR-383-5p were reduced in blood and peri-infarct tissue from the middle cerebral artery occlusion (MCAO) model at 1, 3, and 6 hours of recanalization time. n = 6 rats per group. **(B)** miR-383-5p agomir treatment increased the expression of miR-383-5p in blood and peri-infarct tissue from the MCAO model. n = 5 rats per group. Data are expressed as mean ± standard deviation and were analyzed using one-way ANOVA with Bonferroni *post hoc* correction. Data are from 5 to 6 rats per group (biological replicates). For each PCR experiment, technical triplicates were averaged. *P < 0.05; **P < 0.01; ***P < 0.001 vs. sham group (or vs. MCAO group). I/R indicates ischemia-reperfusion.

Analysis revealed that miR-383-5p was downregulated in the blood and peri-infarct tissue of MCAO rats after mechanical recanalization at 1, 3, and 6 hours (**Figure 1A**). Upregulated miR-383-5p levels were detected in both blood and peri-infarct tissue after the intravenous injection of miR-383-5p agomir when compared with the ischemia-reperfusion group (**Figure 1B**).

### Increased levels of miR-383-5p suppressed brain edema, infarct volume, hemorrhage, and preserved BBB integrity with improved acute outcomes

**Figure 2:**
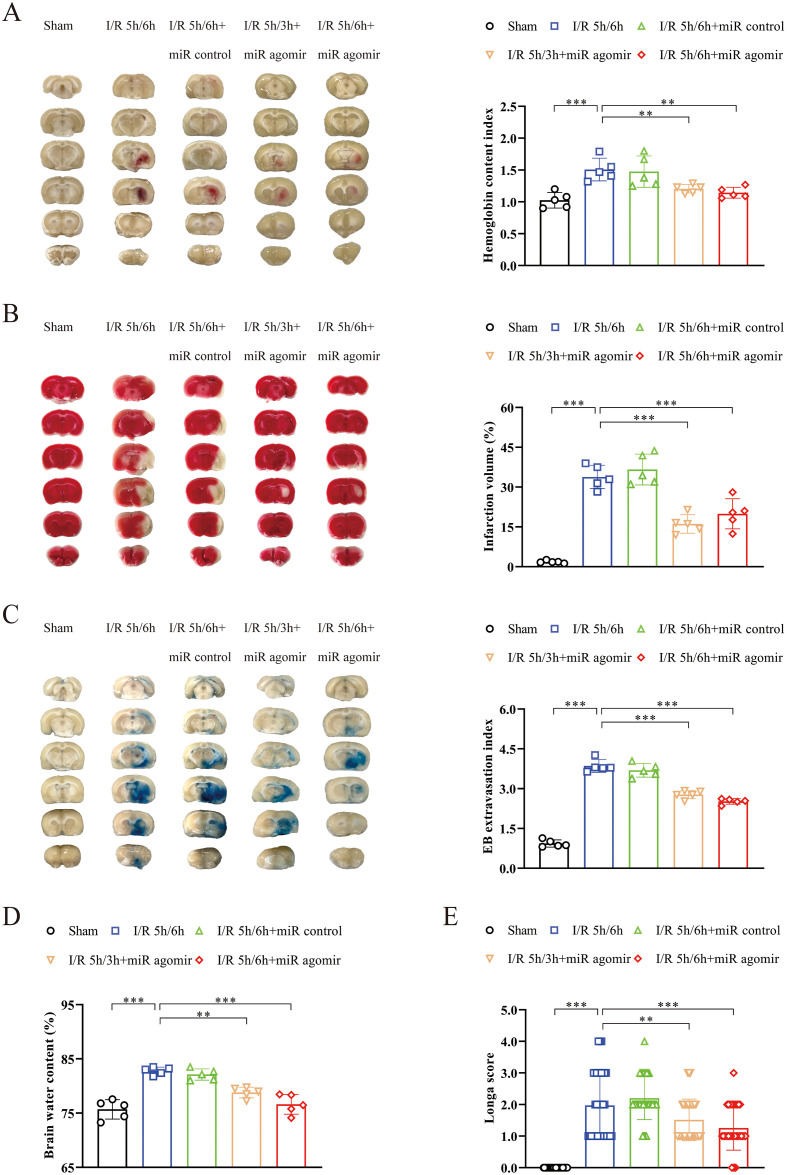
Administration of a miR-383-5p agomir reduced hemorrhage, infarct volume, brain edema, and blood-brain barrier disruption and improved acute outcome in a hyperglycemia-associated, recanalization-induced hemorrhagic transformation model. **(A)** Hemoglobin content index for the assessment of hemorrhage severity at 3 hours and 6 hours after recanalization with 5-hour ischemia. n = 5 rats per group. **(B)** TTC staining of brain slices and quantification for infarct volume at 3 hours and 6 hours after recanalization with 5-hour ischemia. n = 5 rats per group. **(C)** Blood-brain barrier permeability in the ischemic hemisphere, as demonstrated by Evans blue staining. n = 5 rats per group. **(D)** Brain water content as an assessment of brain edema. n = 5 rats per group. **(E)** The miR-383-5p agomir improved acute outcomes with a lower neurological deficit score. n = 5 rats per group. Data are expressed as mean ± standard deviation and were analyzed by one-way ANOVA with Bonferroni *post hoc* correction. Data are from 5 rats per group (biological replicates). For each measurement of hemoglobin content index and Evans blue extravasation index, technical triplicates were averaged. *P < 0.05; **P < 0.01; ***P < 0.001 vs. MCAO group. I/R indicates ischemia-reperfusion.

Treatment with the miR-383-5p agomir treatment reduced the severity of hemorrhage, as demonstrated by hemoglobin content index assessment (1.2 versus 1.5, p=0.006, at 3 hours; 1.1 versus 1.5, p=0.001, at 6 hours) (**Figure 2A**), and reduced infarct volume, as demonstrated by TTC staining (16.1% versus 33.8%, p<0.001, at 3 hours; 19.9% versus 33.8%, p<0.001, at 6 hours) (**Figure 2B**), compared with MCAO rats receiving mechanical recanalization. The marked EB extravasation after ischemia-reperfusion was significantly reduced after treatment with the miR-383-5p agomir (2.8 versus 3.9, p<0.001, at 3 hours; 2.5 versus 3.9, p<0.001, at 6 hours), suggesting that BBB integrity was protected by increased levels of miR-383-5p (**Figure 2C**). Rats receiving the miR-383-5p agomir exhibited alleviation of water content in the brain (78.8% versus 82.8%, p=0.001, at 3 hours; 76.6% versus 82.8%, p<0.001, at 6 hours) (**Figure 2D**), and showed a lower neurological deficit score than those in the ischemia-reperfusion group (1.5 versus 2.0, p<0.001, at 3 hours; 1.3 versus 2.0, p<0.001, at 6 hours) (**Figure 2E**).

### Increased levels of miR-383-5p reduced NOX4 overexpression in the brain microvascular endothelial cells, neurons, and astrocytes in the brains of the mechanical recanalization-induced HT model

**Figure 3:**
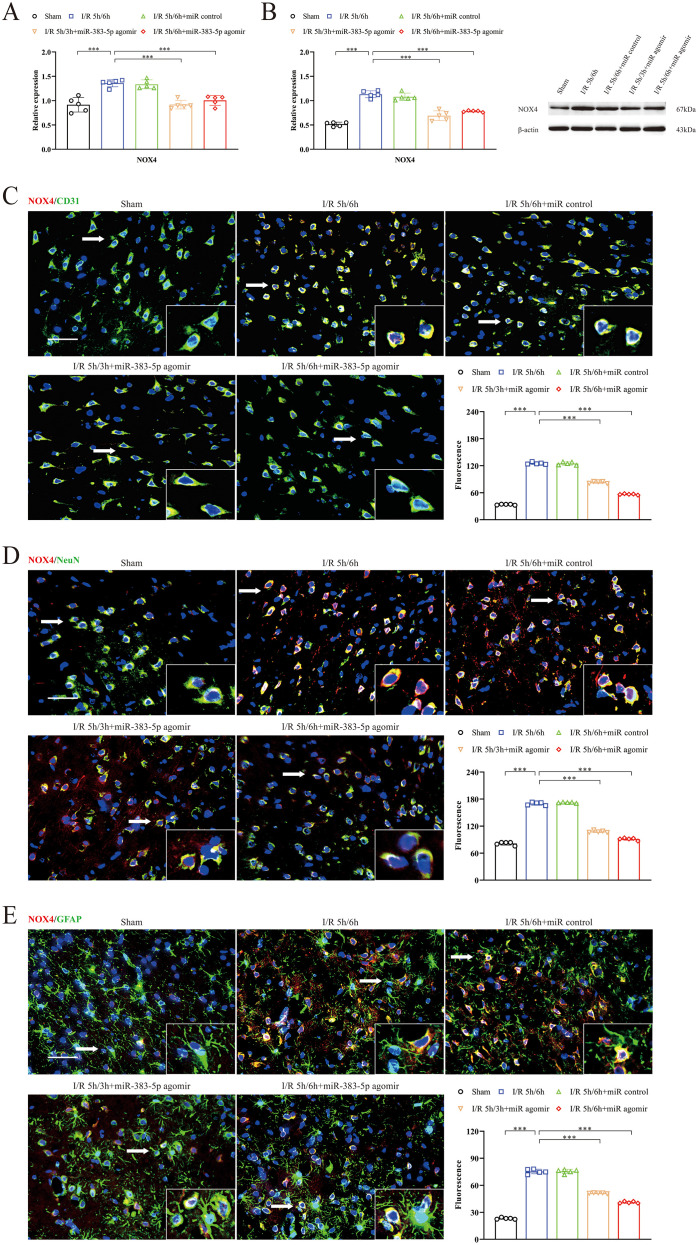
The miR-383-5p agomir protected the peri-infarct region by regulating NADPH oxidase 4 (NOX4) in a hyperglycemia-associated, recanalization-induced hemorrhagic transformation model. **(A, B)** miR-383-5p agomir treatment reduced NOX4 expression in the peri-infarct tissue from the middle cerebral artery occlusion (MCAO) model **(A)** RT-PCR; **(B)** Western blot). n = 5 rats per group. **(C)** miR-383-5p agomir treatment reduced NOX4 (red) overexpression in brain microvascular endothelial cells (green) in peri-infarct tissue, as demonstrated by immunofluorescence staining. n = 5 rats per group. **(D)** miR-383-5p agomir treatment reduced NOX4 (red) overexpression in neurons (green) in peri-infarct tissue, as demonstrated by immunofluorescence staining. n = 5 rats per group. **(E)** miR-383-5p agomir treatment reduced NOX4 (red) overexpression in astrocytes (green) in peri-infarct tissue, as demonstrated by immunofluorescence staining. n = 5 rats per group. Scale bars represent 50 μm. Data are expressed as mean ± standard deviation and were analyzed by one-way ANOVA with Bonferroni *post hoc* correction. Data are from 5 rats per group (biological replicates). For each PCR experiment and immunofluorescence staining image measurement, technical triplicates were averaged. *P < 0.05; **P < 0.01; ***P < 0.001 vs. MCAO group. NeuN indicates neuronal nuclear protein as a marker of neurons. GFAP indicates glial fibrillary acidic protein as a marker of astrocytes. CD31 indicates platelet endothelial cell adhesion molecule-1 as a marker for brain microvascular endothelial cells. I/R indicates ischemia-reperfusion.

Levels of NOX4 mRNA and protein were upregulated in the peri-infarct region of MCAO rats following mechanical recanalization at 3 and 6 hours. This increase was suppressed following the intravenous injection of miR-383-5p agomir (**Figures 3A, B**). As demonstrated by immunohistochemical fluorescent staining, NOX4 expression in the brain microvascular endothelial cells of peri-infarct tissue from the MCAO rats after mechanical recanalization at 6 hours was significantly increased compared with the sham group, whereas miR-383-5p agomir intervention suppressed this increase at 3 hours and 6 hours after mechanical recanalization (**Figure 3C**). Similar changes of NOX4 expression were also detected in neurons and astrocytes within the peri-infarct region. NOX4 expression levels in neurons from the peri-infarct tissue of MCAO rats after mechanical recanalization at 6 hours was significantly increased compared with the sham group, whereas miR-383-5p agomir intervention suppressed this increase at 3 hours and 6 hours after mechanical recanalization (**Figure 3D**). In addition, NOX4 expression in the astrocytes of peri-infarct tissue from MCAO rats after mechanical recanalization at 6 hours was significantly increased compared with the sham group, whereas miR-383-5p agomir intervention suppressed this increase at 3 hours and 6 hours after mechanical recanalization (**Figure 3E**). NOX4 expression in microglia of the peri-infarct tissue from MCAO rats after mechanical recanalization at 6 hours was significantly increased compared with the sham group; miR-383-5p agomir intervention did not suppress this increase at either 3 or 6 hours after mechanical recanalization.

### Increased levels of miR-383-5p reduced ROS overproduction and neuronal apoptosis in the mechanical recanalization-induced model of HT

**Figure 4:**
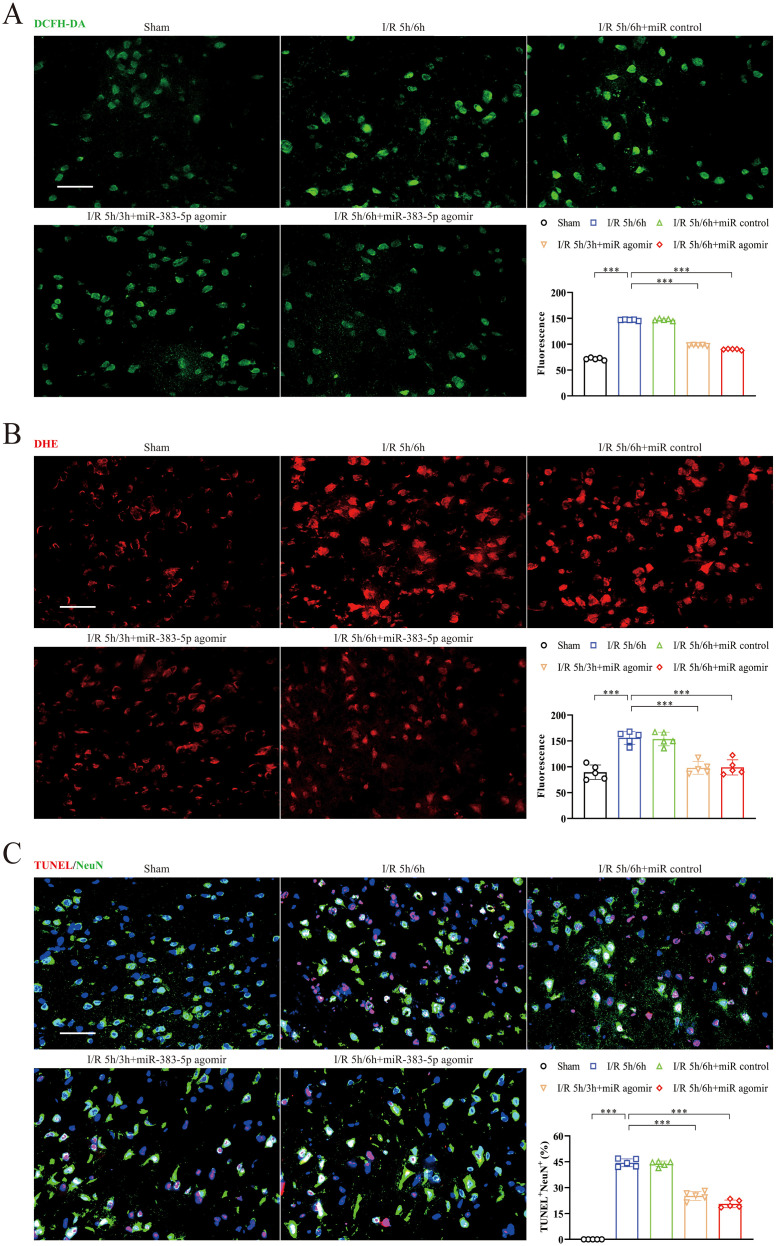
The miR-383-5p agomir protected the peri-infarct region against oxidative damage and apoptosis by regulating the NADPH oxidase 4 (NOX4)/reactive oxygen species (ROS) signaling pathway. **(A)** miR-383-5p agomir treatment reduced ROS overproduction in the peri-infarct region, as quantified by DCFH-DA staining (green). n = 5 rats per group. **(B)** miR-383-5p agomir treatment reduced ROS overproduction in the peri-infarct region, as quantified by DHE staining (red). n = 5 rats per group. **(C)** miR-383-5p agomir treatment reduced neuronal apoptosis in the peri-infarct region, as demonstrated by TUNEL (red) and NeuN (green) immunolabeling. n = 5 rats per group. Scale bars represent 50 μm. Data are expressed as mean ± standard deviation and were analyzed by one-way ANOVA with Bonferroni *post hoc* correction. Data are from 5 rats per group (biological replicates). For fluorescence staining image measurement, technical triplicates were averaged. *P < 0.05; **P < 0.01; ***P < 0.001 vs. MCAO group. DCFH-DA indicates 2’,7’-dichlorodihydrofluorescein diacetate. DHE indicates dihydroethidium. NeuN indicates neuronal nuclear protein as a neuronal marker. TUNEL indicates terminal deoxynucleotidyl transferase dUTP nick end labeling (TUNEL). I/R indicates ischemia-reperfusion.

The overproduction of ROS in the peri-infarction region was demonstrated by DCFH-DA and DHE staining after ischemia-reperfusion; intervention with the miR-383-5p agomir significantly attenuated superoxide anion and hydrogen peroxide production (**Figures 4A, B**). TUNEL-positive neurons, reflecting apoptosis in peri-infarct tissue, were far more abundant after ischemia/reperfusion, although this was reduced following treatment with the miR-383-5p agomir treatment (**Figure 4C**).

### Increased levels of miR-383-5p reduced astrocytic inflammation and preserved BBB-associated proteins

**Figure 5:**
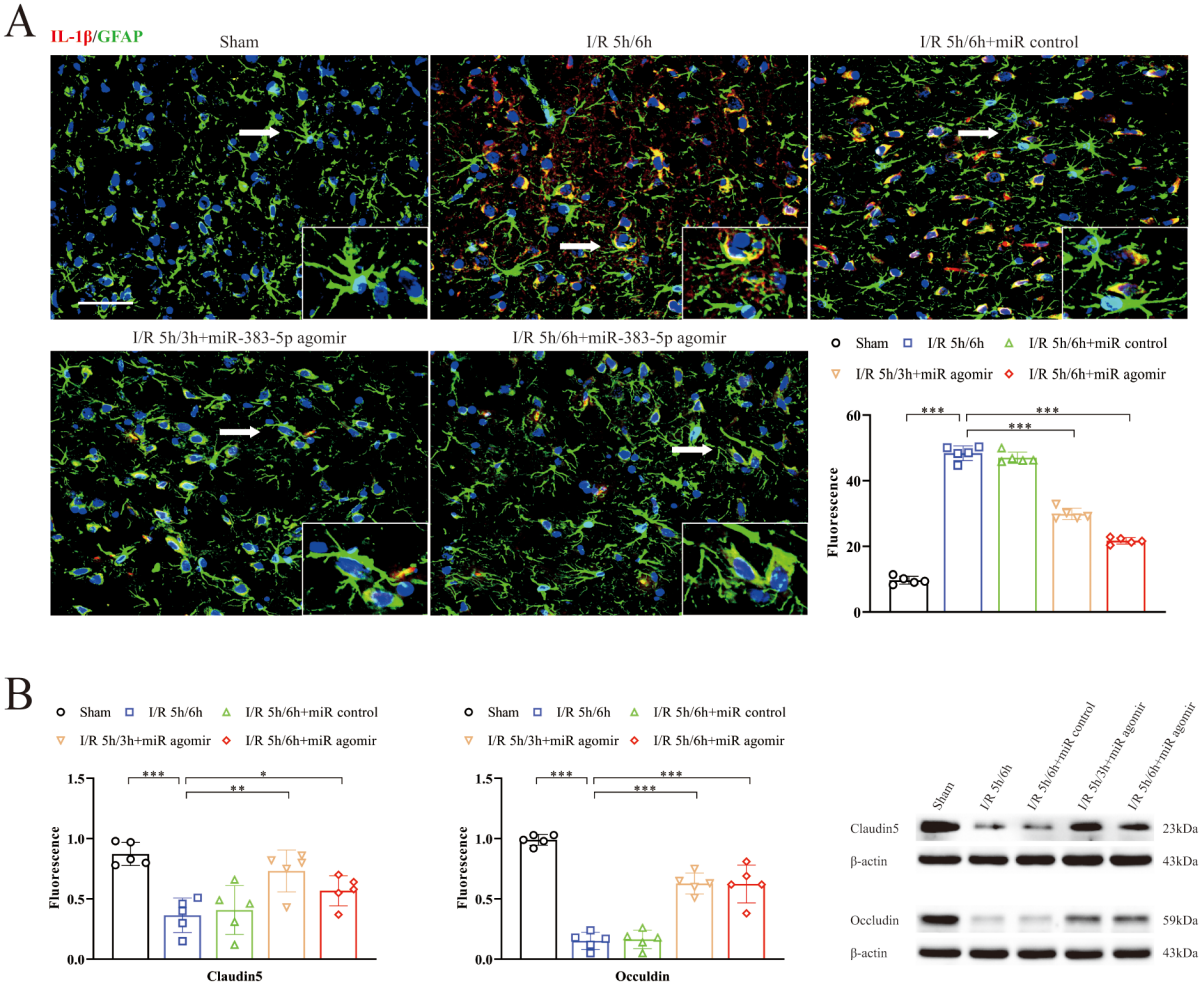
The miR-383-5p agomir reduced astrocytic inflammation and preserved blood-brain barrier-associated proteins in a hyperglycemia-associated, recanalization-induced hemorrhagic transformation model. **(A)** miR-383-5p agomir treatment reduced interleukin 1 beta (IL-1β) (red) overexpression in astrocytes (green) in peri-infarct tissue, as demonstrated by immunofluorescence staining. n = 5 rats per group. **(B)** the miR-383-5p agomir restored the claudin5 and occludin expression in the peri-infarct tissue, as demonstrated by Western blotting. n = 5 rats per group. Scale bars represent 50 μm. Data are expressed as mean ± standard deviation and were analyzed by one-way ANOVA with Bonferroni *post hoc* correction. Data are from 5 rats per group (biological replicates). For immunofluorescence staining image measurement, technical triplicates were averaged. *P < 0.05; **P < 0.01; ***P < 0.001 vs. MCAO group. GFAP indicates glial fibrillary acidic protein as a marker for astrocytes. I/R indicates ischemia-reperfusion.

As demonstrated by immunohistochemical fluorescent staining, IL-1β expression in astrocytes from MCAO rats after mechanical recanalization was significantly increased compared with the sham group; intervention with the miR-383-5p agomir suppressed this increase at 3 and 6 hours after mechanical recanalization (**Figure 5A**).

MiR-383-5p agomir treatment partially restored the expression of the tight junction proteins claudin5 and occludin; both were reduced in the peri-infarct tissue after ischemia-reperfusion (**Figure 5B**).

### NOX4 was identified as the direct target gene for miR-383-5p

**Figure 6:**
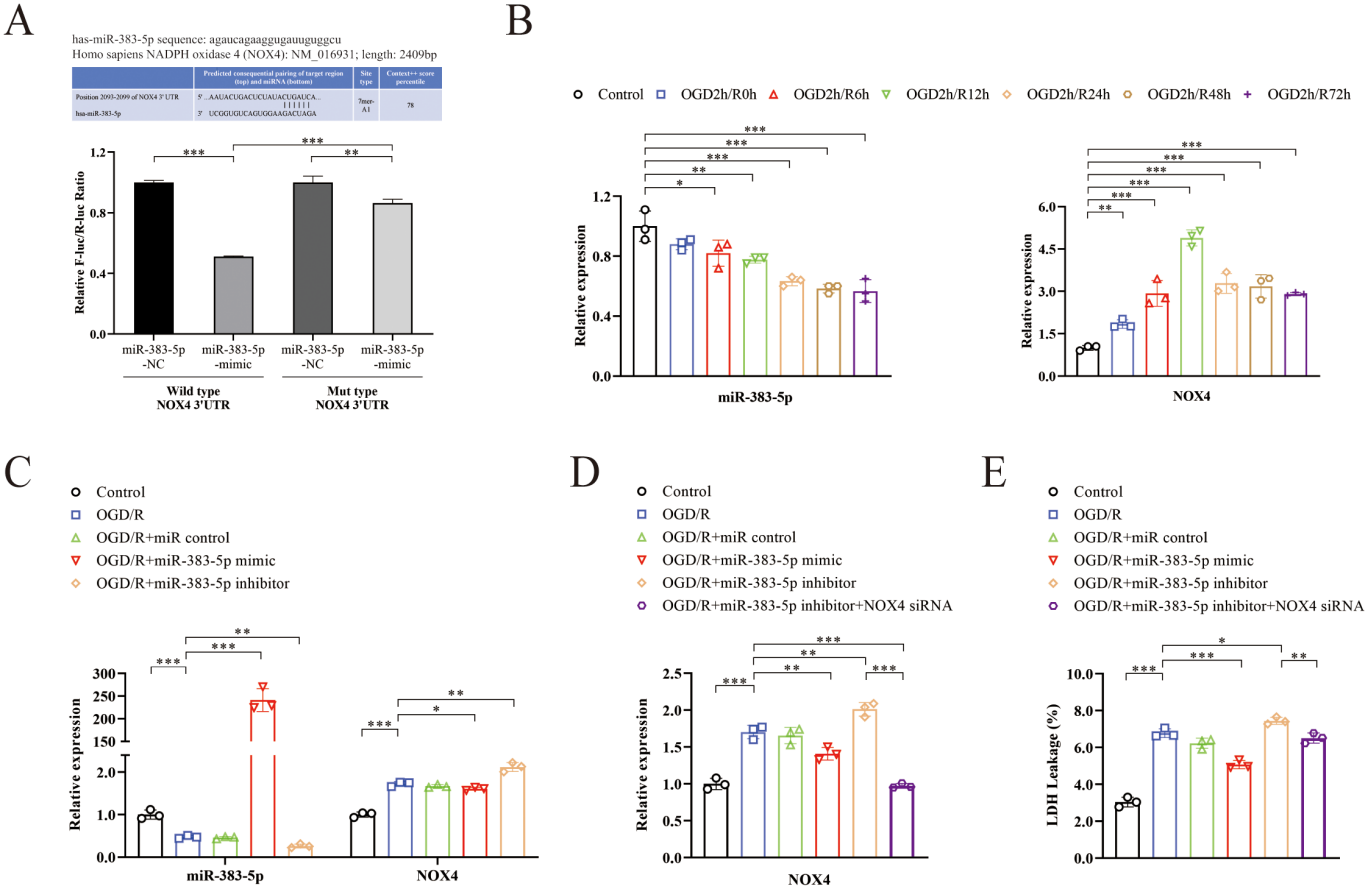
Elevating miR-383-5p levels exerted a neuroprotective role in the oxygen-glucose deprivation/reoxygenation (OGD/R) model of neurons by suppressing NADPH oxidase 4 (NOX4) overexpression. **(A)** NOX4 was identified as the direct target gene of miR-383-5p, as determined by a dual-luciferase reporter assay. **(B)** miR-383-5p expression decreased, and NOX4 expression increased following OGD/R at 6, 12, 24, 48, and 72 hours. **(C)** miR-383-5p mimics intervention increased miR-383-5p expression and decreased NOX4 upregulation. **(D)** MiR-383-5p inhibitor intensified NOX4 upregulation. The combination of a miR-383-5p inhibitor and NOX4 siRNA reversed NOX4 upregulation. **(E)** miR-383-5p mimics inhibited lactate dehydrogenase release from neurons. Data are expressed as mean ± standard deviation. Statistical analysis was performed using the Student’s t-test for panel **(A)** and one-way ANOVA with Bonferroni *post hoc* correction for panels **(B–E)** Data are from three independent experiments (biological replicates). For each experiment, technical triplicates were averaged. *P < 0.05; **P < 0.01; ***P < 0.001 vs. control group (or vs. OGD/R group).

Dual-luciferase reporter assays, combined with bioinformatic predictions, confirmed that NOX4 is a direct target of miR-383-5p. The inhibitory effect of miR-383-5p mimics on the target gene NOX4 3’UTR was detected by dual-luciferase reporter assays. Site-specific mutagenesis technology determined the site of interaction between miR-383-5p and the NOX4 3´UTR. MiR-383-5p mimics significantly inhibited the luciferase activity expressed in tandem with the wild-type NOX4 3´UTR and partially inhibited the luciferase activity expressed in tandem with the mutant NOX4 3´UTR; however, the inhibitory ability was partially weakened (**Figure 6A**).

### Increased miR-383-5p inhibited NOX4 overexpression and alleviated neuronal injury following OGD/R

Reduced miR-383-5p expression was detected in neurons following OGD/R, concomitant with increased expression levels of NOX4 (**Figure 6B**). Following miR-383-5p mimics intervention, NOX4 upregulation in neurons was reduced. Intervention with a miR-383-5p inhibitor intensified the upregulation of NOX4 (**Figure 6C**). NOX4 siRNA, combined with a miR-383-5p inhibitor, alleviated the NOX4 upregulation, suggesting that miR-383-5p regulated NOX4 in OGD/R-treated neurons (**Figure 6D**). LDH release from OGD/R-treated neurons was inhibited by miR-383-5p mimics, but was exacerbated by the miR-383-5p inhibitor (**Figure 6E**).

### Increased levels of miR-383-5p inhibited ROS by regulating NOX4 in the OGD/R model in neurons

**Figure 7:**
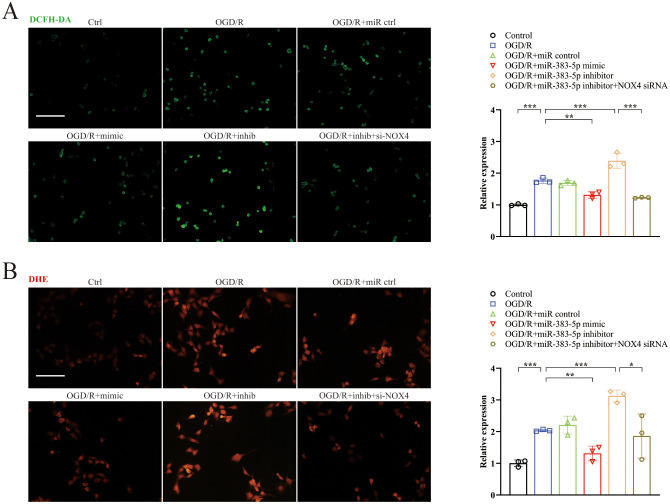
Increased levels of miR-383-5p reduce reactive oxygen species overproduction by suppressing NADPH oxidase 4 (NOX4) in the neuronal oxygen-glucose deprivation/reoxygenation (OGD/R) model. Detection of hydrogen peroxide by DCFH-DA (green) **(A)** and superoxide anion by DHE (red) **(B)** in the neuronal OGD/R model treated with miR-383-5p mimic and inhibitor, and NOX4 siRNA. Scale bars represent 50 μm. Data are expressed as mean ± standard deviation and were analyzed by one-way ANOVA with Bonferroni *post hoc* correction. Data are from three independent experiments (biological replicates). For each experiment, technical triplicates were averaged. *P < 0.05; **P < 0.01; ***P < 0.001 vs. OGD/R group.

Intervention with miR-383-5p mimics inhibited the increase in ROS (hydrogen peroxide and superoxide anion) observed in neurons induced by OGD/R. Furthermore, treatment with the miR-383-5p inhibitor intensified ROS overproduction following OGD/R. NOX4 siRNA combined with the miR-383-5p inhibitor attenuated ROS upregulation, suggesting that miR-383-5p directly regulated NOX4 to attenuate the overproduction of ROS (**Figure 7**).

## Discussion

Analysis revealed that downregulation of miR-383-5p and upregulation of NOX4 in the penumbra are associated with hemorrhage after reperfusion intervention in AIS, as demonstrated in a rat MCAO model after mechanical recanalization, and in the neuronal OGD/R model. Increased miR-383-5p expression was shown to inhibit ROS overproduction, neuronal apoptosis, and astrocytic inflammation by suppressing the upregulation of NOX4 (the direct target gene) in brain microvascular endothelial cells, neurons, and astrocytes in the peri-infarct region, and to preserve junction proteins associated with the BBB. This process reduced brain edema, infarction volume, hemorrhage, and BBB breakdown, thereby alleviating acute neurological deficits assessed at 3 and 6 hours after recanalization. Collectively, these findings provide new evidence that miR-383-5p/NOX4/ROS signaling axis is a promising target for reducing HT in AIS, beyond previous studies that have shown the individual roles of miR-383-5p or NOX4 in cerebral ischemia (6–8, 17).

As one of the most abundant neuron-associated microRNAs, miR-383 is implicated in various neurological disorders by regulating apoptosis, proliferation, and oxidative stress. MiR-383-5p expression was previously shown to be reduced in blood samples from patients with Alzheimer’s disease in comparison with healthy volunteers. The upregulated miR-383-5p attenuates Aβ-triggered neuronal apoptosis and oxidative stress injury by reducing the expression of kinesin family member 1B, a direct target gene (18). A previous study using neuronal OGD/R and MCAO models demonstrated that miR-383-5p protects against ischemic brain injury by regulating the activity of histone deacetylase 9 (HDAC9). The CCCTC-binding factor is known to downregulate miR-383-5p and upregulate HDAC9 expression in cerebral ischemia, thereby promoting neuronal apoptosis and aggravating neuronal damage induced by endoplasmic reticulum stress (17). These findings support our results in that increased levels of miR-383-5p could provide a neurovascular protective effect to alleviate HT after mechanical recanalization in AIS. Notably, miR-383-5p and miR-383-3p isoforms may perform different roles in stroke-induced brain injury. The activation of microglia following intracerebral hemorrhage leads to the transfer of miR-383-3p into neurons, which subsequently negatively regulates the expression of activating transcription factor 4, thereby promoting neuronal necroptosis (19). In the microglia OGD/R model, miR-383-3p was shown to directly target the downstream src homology 2 domain-containing phosphatase 2 (SHP2) to regulate nucleotide-binding oligomerization domain-like receptor protein 3 (NLRP3) and associated cytokines. Transcranial focused ultrasound stimulation was also shown to exert a neuroprotective effect in ischemic stroke by suppressing NLRP3 via the Nespas/miR-383-3p/SHP2 pathway (20).

Our analyses showed that elevated levels of miR-383-5p exerted neurovascular protective effects by regulating its direct target gene, NOX4. In humans, seven NOX isoforms are known to produce superoxide anions and hydrogen peroxide to maintain a balance in NOX-ROS signaling under normal physiological conditions. In cases of cerebral ischemia and intracerebral hemorrhage, a disruption in redox balance, caused by the excessive induction of NOX, initiates a high level of ROS that exacerbates injury to neurovascular cells (5). NOX4-focused ROS signaling can be triggered by cerebral ischemia, resulting in oxidative stress damage and neuronal apoptosis. Treatment with an NOX4 isoform-specific inhibitor or the genetic inhibition of NOX4 has been shown to reduce NOX4 overexpression and excessive ROS generation, thereby protecting against ischemia-induced neuronal damage (6, 21). NOX4 is also involved in oxidative stress injury and neuronal ferroptosis following intracerebral hemorrhage, making NOX4 a neuroprotective target for alleviating the burden of hemorrhage and secondary brain injury (22, 23). Following cerebral ischemia, neuronal NOX4 contributes to neuronal damage, whereas endothelial NOX4 disrupts the integrity of the BBB (24). MiR-100-5p-loaded exosomes were previously shown to alleviate oxidative stress damage by targeting NOX4 in a brain endothelial cell OGD/R model; they also reduced infarct volume and improved functional outcomes in the MCAO model (8). In our previous study, we detected the reduced levels of miR-383-5p in neuronal and astrocytic models of OGD/R; however, the expression of miR-383-5p did not change in an OGD/R model of cerebral endothelial cells, pericytes, and microglia cells (12). In this current study, involving an animal model, we detected excessive levels of ROS generated by NOX4 upregulation in neurons, astrocytes, and brain microvascular endothelial cells. These changes were due to the reduced levels of miR-383-5p following ischemia-reperfusion injury, which can trigger neurovascular unit damage and BBB disruption by downregulating junctional complexes between endothelial cells. Treatment with a miR-383-5p mimic inhibited neuronal, astrocytic, and endothelial NOX4 overexpression in the peri-infarct region and then reduced excessive ROS production. These processes resulted in the restoration of tight junction proteins (claudin5 and occludin) in the peri-infarct region, and a reduction in HT in our animal model. Previous studies reported that several microRNAs can regulate NOX4 expression in various disease models, including chronic oxidative stress, vascular injury, acute ischemic injury, and neurodegenerative conditions (8, 25–27). In the present study, we identified miR-383-5p as a direct regulator of NOX4, specifically in reperfusion-induced HT. The uniqueness of miR-383-5p lies in its ability to reduce the severity of HT, preserve the integrity of the BBB, and improve acute outcomes in a model mimicking clinical endovascular thrombectomy for acute stroke patients at high risk of HT.

Previous studies also reported that NOX4 plays a crucial role in the inflammatory mechanisms underlying central nervous system diseases in brain microvascular endothelial cells, astrocytes, and neurons. NOX4 is activated in brain microvascular endothelial cells after cerebral ischemia, leading to excess ROS that directly induces apoptosis and disrupts the BBB, thereby triggering and exacerbating secondary brain injury (28). In astrocytes, NOX4 is upregulated during cerebral ischemia-reperfusion injury and produces an excess of ROS to activate the toll-like receptor 4/NF-κB inflammatory pathway. In addition, astrocytic NOX4 promotes the release of pro-inflammatory factors, such as IL-1β, through the NLRP3 inflammasome. These effects transform ischemic stress into destructive neuroinflammation, leading to neuronal death and damage to the BBB (13, 29–31). Neurons are not only adversely affected by the inflammatory factors released by astrocytes, but also, importantly, neuronal NOX4 acts as a key executor of ferroptosis, directly amplifying the inflammatory response through lipid peroxidation (14). These findings further support the discovery of this study, which demonstrated that miR-383-5p regulates the direct target gene NOX4 in brain microvascular endothelial cells, astrocytes, and neurons to mediate inflammatory cascades, thereby mitigating HT after mechanical recanalization in AIS.

The study has several limitations that need to be considered. For example, this study was designed as an acute, mechanistic, and proof-of-concept study to test whether the administration of miR-383-5p before recanalization could reduce early HT-associated injury. Therefore, we are unable to determine whether the protective effects of miR-383-5p persist, diminish, or are followed by delayed exacerbation. The effect of increased miR-383-5p levels on long-term outcomes was not investigated, and neurobehavioral assessments were not conducted beyond the 6-hour time window. The model used herein, with hyperglycemic conditions and severe cerebral ischemia, can create more severe cases of HT, including parenchymal hematoma, as reported in our previous studies (7, 12, 15, 16). Our model represents a high-risk subgroup rather than the average thrombectomy patient. However, in the recent clinical setting, more acute stroke patients with a large ischemic core and up to 12–24 hours after ischemia have been endovascularly treated and then developed asymptomatic HT (1, 4, 32). In addition, hyperglycemia can independently induce oxidative stress and disrupt the BBB. Therefore, we cannot rule out that at least a component of the protective effect of miR-383-5p was due to the counteraction of hyperglycemia-induced injury. Our current findings may differ in normoglycemic models; therefore, future studies need to utilize normoglycemic animals to assess generalizability.

We assessed BBB integrity after miR-383-5p intervention by Evans blue leakage and the expression of claudin5 and occludin. However, BBB dysfunction is a complex process involving multiple components beyond these markers. Additional BBB markers (zonula occludens-1, matrix metalloproteases) and ultrastructural analysis may be needed to validate these effects fully. Importantly, we measured only total protein levels of claudin5 and occludin in the peri-infarct tissue and did not perform co-localization with brain microvascular endothelial cell markers to confirm their structural localization at the microvascular wall. Further, peripheral immune cell infiltration is an important consequence of BBB disruption. We did not assess whether miR-383-5p agomir inhibits leukocyte infiltration into ischemic tissues using flow cytometry or immunohistochemistry. Third, we did not establish an *in vitro* co-culture system of brain microvascular endothelial cells and astrocytes in Transwell chambers to assess endothelial barrier integrity following OGD/R. Such a system would allow direct measurement of transendothelial electrical resistance and permeability. While our *in vivo* data show reduced Evans blue extravasation and preserved tight junction proteins, both of which are consistent with improved BBB integrity, direct evidence of tight junction localization, reduced immune cell infiltration, and *in vitro* barrier function remains lacking. Future studies warrant clarification of these limitations using immunofluorescence to assess tight junction colocalization, immunophenotyping to assess immune cell infiltration, and co-culture models to assess endothelial barriers.

We confirmed that NOX4 is a direct target of miR-383-5p using luciferase reporter assays and showed that miR-383-5p agomir reduces NOX4 expression *in vivo*; however, we did not perform *in vivo* rescue experiments (e.g., NOX4 overexpression or knockdown in the presence of miR-383-5p modulation). Therefore, the protective effects of miR-383-5p mediated exclusively through NOX4 cannot be definitively established. We observed that miR-383-5p agomir suppressed NOX4 upregulation in neurons, astrocytes, and endothelial cells, but not in microglia. Neurons are particularly vulnerable to oxidative stress due to their high metabolic demand and limited antioxidant capacity. NOX4-derived ROS plays a critical role in neuronal apoptosis following ischemia-reperfusion. NOX4 suppression by miR-383-5p in neurons likely contributes substantially to reduced neuronal apoptosis and improved acute outcomes. In contrast, microglial NOX4 upregulation occurs within the 6-hour observation window; however, lower miR-383-5p agomir uptake efficiency may occur in microglia, as well as cell-type-specific transcriptional regulation of NOX4 that overrides miR-383-5p-mediated suppression. Future studies using cell-type-specific modulation of NOX4 are needed to clarify the functional role of microglial NOX4 in HT.

We observed that miR-383-5p agomir reduced IL-1β overexpression in astrocytes. However, the underlying mechanism was not experimentally validated. We did not directly assess other downstream inflammatory pathways beyond IL-1β, such as NF-κB activation, NLRP3 inflammasome, or ferroptosis markers. Matrix metalloproteases (MMPs) play an important role in BBB disruption. However, we did not perform MMP activity or expression assays. We speculated that miR-383-5p may reduce MMP expression by inhibiting ROS to preserve tight junction proteins. Direct evidence linking miR-383-5p, NOX4/ROS, and MMP regulation is lacking. Other target genes directly regulated by miR-383-5p function as neurovascular protectors to alleviate hemorrhage after mechanical recanalization, which warrants further clarification through RNA sequencing in combination with animal studies and neurovascular cell OGD/R models. Finally, a formal power calculation was not performed before the study. Although our sample sizes were based on previous publications in the field, the lack of prospective power analysis is a methodological limitation.

With regard to clinical translation, measuring circulating miR-383-5p levels in patient plasma or cerebrospinal fluid could serve as a potential biomarker. Baseline miR-383-5p levels may help to identify patients at a higher risk of HT, whereas post-treatment levels can be used to monitor target engagement or predict therapeutic response (9). Such biomarker validation would require prospective clinical cohort studies in patients undergoing endovascular thrombectomy. In the present study, we administered the miR-383-5p agomir 30 minutes prior to retrieving the intraluminal filament to ensure drug delivery before reperfusion injury onset. Although pre-recanalization administration is not clinically feasible for most patients undergoing endovascular thrombectomy, we selected this timing for mechanistic proof-of-concept. Intra-arterial delivery during thrombectomy or immediate post-recanalization loading would need to be tested for clinical translation (33, 34). MicroRNA agomirs have a short half-life; lipid nanoparticle encapsulation or chemical modifications may improve stability (35, 36). Although ischemia-reperfusion conditions compromise BBB integrity, optimizing brain delivery via receptor-mediated transcytosis or focused ultrasound may be beneficial. Potential risks include immunogenicity, hepatotoxicity, and thrombocytopenia, which require evaluation in large-animal models. Thus, our findings provide mechanistic support for the miR-383-5p/NOX4 axis, but significant preclinical development remains before clinical testing.

## Conclusions

Our analyses highlight the neurovascular protective role of miR-383-5p in preventing hemorrhagic transformation. Analysis revealed that miR-383-5p alleviated oxidative stress injury, neuronal apoptosis, and inflammation, and preserved BBB integrity by targeting NOX4. Thus, miR-383-5p represents a potential target for intervention to reduce hemorrhage and improve outcomes during the acute phase of recanalization after endovascular treatment for AIS. Long-term outcomes following miR-383-5p intervention remain to be further assessed.

## Data Availability

The datasets presented in this study can be found in online repositories. The names of the repository/repositories and accession number(s) can be found in the article/supplementary material.
